# Persistent Cell-Autonomous Circadian Oscillations in Fibroblasts Revealed by Six-Week Single-Cell Imaging of PER2::LUC Bioluminescence

**DOI:** 10.1371/journal.pone.0033334

**Published:** 2012-03-29

**Authors:** Tanya L. Leise, Connie W. Wang, Paula J. Gitis, David K. Welsh

**Affiliations:** 1 Department of Mathematics, Amherst College, Amherst, Massachusetts, United States of America; 2 Department of Psychiatry and Center for Chronobiology, University of California San Diego, La Jolla, California, United States of America; 3 Veterans Affairs San Diego Healthcare System, San Diego, California, United States of America; Vanderbilt University, United States of America

## Abstract

Biological oscillators naturally exhibit stochastic fluctuations in period and amplitude due to the random nature of molecular reactions. Accurately measuring the precision of noisy oscillators and the heterogeneity in period and strength of rhythmicity across a population of cells requires single-cell recordings of sufficient length to fully represent the variability of oscillations. We found persistent, independent circadian oscillations of clock gene expression in 6-week-long bioluminescence recordings of 80 primary fibroblast cells dissociated from PER2::LUC mice and kept in vitro for 6 months. Due to the stochastic nature of rhythmicity, the proportion of cells appearing rhythmic increases with the length of interval examined, with 100% of cells found to be rhythmic when using 3-week windows. Mean period and amplitude are remarkably stable throughout the 6-week recordings, with precision improving over time. For individual cells, precision of period and amplitude are correlated with cell size and rhythm amplitude, but not with period, and period exhibits much less cycle-to-cycle variability (CV 7.3%) than does amplitude (CV 37%). The time series are long enough to distinguish stochastic fluctuations within each cell from differences among cells, and we conclude that the cells do exhibit significant heterogeneity in period and strength of rhythmicity, which we measure using a novel statistical metric. Furthermore, stochastic modeling suggests that these single-cell clocks operate near a Hopf bifurcation, such that intrinsic noise enhances the oscillations by minimizing period variability and sustaining amplitude.

## Introduction

Circadian (ca. 24 h) clocks are intracellular timekeeping devices found in organisms from cyanobacteria to humans [1]. These clocks orchestrate daily temporal programs of physiology and behavior, anticipating environmental light/dark transitions and persisting even under constant conditions.

In mammals, circadian timing is organized hierarchically [2]. The primary pacemaker in the brain, the suprachiasmatic nucleus (SCN), is synchronized to the day/night cycle by photic input from the retina, and in turn synchronizes a multitude of subsidiary oscillators throughout the body. Although tissue organization and cellular interactions are important for clock function, particularly in the SCN, individual cells such as SCN neurons or fibroblasts contain autonomous circadian clocks [3]. Within each cell, BMAL1/CLOCK heterodimers activate transcription of *Period (Per)* and *Cryptochrome (Cry)* genes [4]. After delays associated with transcription, translation, formation of molecular complexes, and nuclear translocation, the products of *Per* and *Cry* genes feed back to inhibit transcription of their own genes. After several hours, the inhibition is relieved by protein turnover, allowing the cycle to begin anew.

Precise daily timing of physiological events relative to one another or to environmental events has great adaptive value [5]. Thus, the stability and precision of circadian clocks is of great importance to cells and organisms. Resistance of the clock to environmental and genetic perturbations is enhanced by both intracellular and intercellular mechanisms [6]. Even under constant conditions in isogenic cells, however, the precision of circadian clocks as transcriptional-translational feedback loops is limited by the inherent stochasticity of gene expression [7], [8], [9].

Individual SCN neurons dispersed in culture are independent circadian oscillators, sufficiently stable to generate circadian rhythms of neuronal firing for at least 6 weeks on multielectrode arrays, but exhibit a range of circadian periods (24.35±1.20 h, mean±SD) [10]. The stability and precision of these cellular oscillators are improved substantially by coupling within the SCN multioscillator system, when SCN tissue organization is preserved in vivo or in slice preparations. Specifically, gene expression rhythms in SCN slices measured using bioluminescent reporters can persist for well over a year [11], are resistant to genetic perturbations causing loss of rhythmicity in most single cells [12], and exhibit ∼10-fold less cycle-to-cycle variability in period than dispersed SCN neurons [13]. Herzog et al. [13] compared firing rate rhythms of mouse SCN neurons with clock gene expression rhythms of SCN explants and whole animal locomotor activity rhythms, observing that single cell oscillators are relatively sloppy (median cycle-to-cycle SD of period = 2.07 hrs, or 8.8% of the period), and that precision increases with level of tissue organization. In that study, single cell period data consisted of 6 consecutive peak-to-peak times in multielectrode array recordings of 23 dissociated SCN neurons.

Cells outside the SCN are also independent circadian oscillators, as demonstrated in fibroblasts using fluorescent or bioluminescent reporters of clock gene expression [14], [15]. After serum shock or medium change, cell population rhythms decline in amplitude and eventually damp out, but this is due to gradual desynchronization of undamped single-cell oscillations, as observed directly by PER2::LUC bioluminescence imaging [14]. Like SCN neurons, individual fibroblasts exhibit strong, independent oscillations, with a range of circadian periods (25.65±1.40 hrs, mean ± SD), for at least 11 days [14].

Carr and Whitmore [16] examined variability of circadian oscillations in zebrafish embryonic cell lines transfected with a *zfperiod4-luciferase* reporter construct. Single zebrafish cells continue to oscillate after months in constant conditions, similar to mammalian fibroblasts [14], [15]. The zebrafish cells are directly photosensitive, can be synchronized by a light/dark cycle, and exhibit reduced fluctuations in period immediately following release from a light/dark cycle (compared to cells in long-term constant darkness). For this analysis, Carr and Whitmore calculated daily period values from sliding 2-day windows, in 6-day DD recordings of 40 cells, but they did not quantify the cycle-to-cycle variability. Single-cell rhythms of gene expression have also been reported in cyanobacteria, and found to be extremely precise based on 5–6 day recordings [17].

To better characterize the stability and precision of mammalian single-cell circadian oscillators, we performed extended bioluminescence imaging of fibroblasts from PER2::LUC mice. We find that the intracellular mammalian circadian clock is a noisy oscillator that exhibits significant stochastic fluctuations, but with much less variability in period than in amplitude. With sufficiently long recordings (≥2–3 weeks) we find that 100% of cells are rhythmic, whereas shorter intervals can give the misleading impression of arrhythmicity due to stochastic variability in amplitude. Discriminating the variability due to stochastic fluctuations from that due to heterogeneity in the population revealed significant differences in period, amplitude, and strength of rhythmicity among cells, which may be attributed to epigenetic changes, differences in cell size, and other factors. To further analyze the stochastic properties of the cellular oscillators, we fit the fibroblast data to a simple limit-cycle model to assess whether the oscillations appear to be self-sustained or noise-induced. We find that the majority of fibroblast cells are self-sustained oscillators running near a Hopf bifurcation, with parameter values in a range for which the intrinsic noise minimizes period variability, so that noise can enhance the steadiness of these molecular clocks.

## Results

### All cells are rhythmic, but many appear non-rhythmic over short intervals

**Figure 1:**
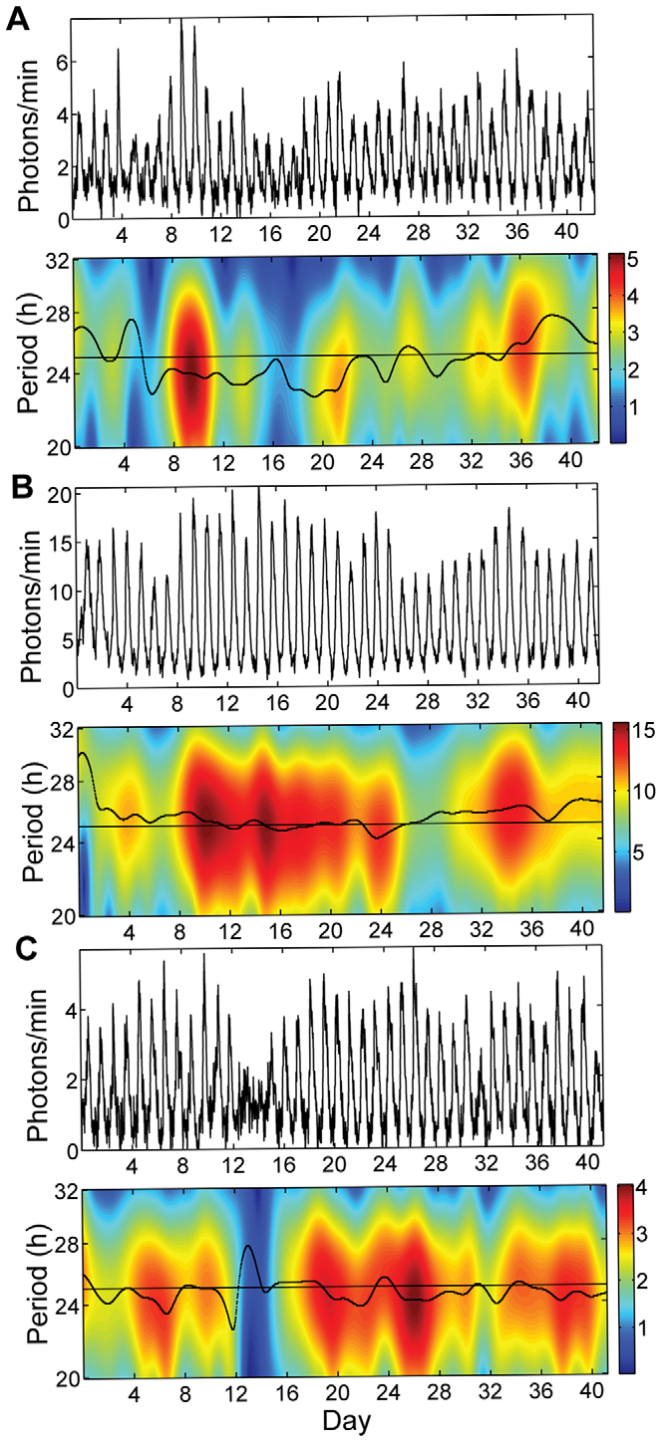
Examples of fibroblast PER2::LUC recordings. The time series for each example is shown above the corresponding analytic wavelet transform (AWT) to illustrate the variability over time in period and amplitude. Period as a function of time is indicated by the black ridge curve, while amplitude is indicated by the color scale (in photons/min). A line at period 25 h is included for reference. (A) Typical cell #11, cell area 1.12×10^4^ µm^2^, period 24.3 h with CV 0.067, amplitude 2.94 photons/min with CV 0.35. Note that red in the AWT corresponds to cycles with high amplitude, yellow those with moderate amplitude, and blue those with low amplitude. Period variability is indicated by the black ridge curve moving up and down over time. (B) Large cell #25, cell area 1.74×10^4^ µm^2^, period 25.3 h with CV 0.030, amplitude 12.2 photons/min with CV 0.23, exhibiting steady rhythms, with both amplitude and period varying less than in (A). (C) Cell #36 with strong oscillations except for a pause on days 13–14 (reflected by the blue region of the AWT), cell area 8.36×10^3^ µm^2^, period 24.8 h with CV 0.055, amplitude 2.93 photons/min with CV 0.27.

**Figure 2:**
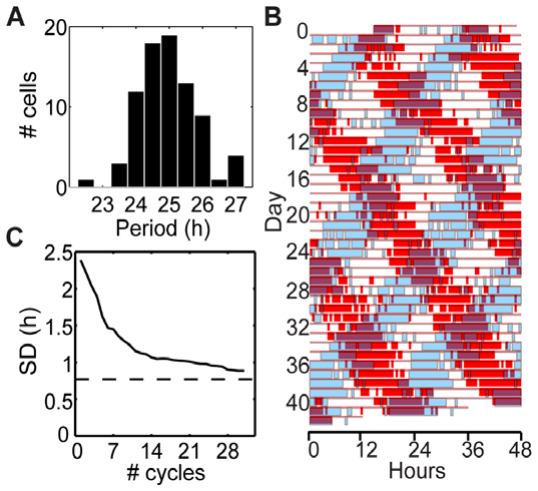
Analysis of cell periods. (A) Histogram of cell periods (mean peak-to-peak times). (B) Raster plot showing two cells with clearly different periods. In the raster plot, time of day is plotted left to right and successive days down the page, such that vertically adjacent points are 24 h apart. Each row is extended to 48 h, duplicating data in the next row, so that patterns crossing midnight can be appreciated. Thick bars designate times when the luminescence for a cell was above the mean for each row. Cell #66 with period 25.5 h is plotted in red; cell #68 with period 22.5 h is plotted in blue. Due to different circadian periods, the two cells' phase relationship changes over time. (C) Standard deviation in period over the population of cells as a function of the number of cycles used for period determination. Here period for each cell is calculated as the mean of peak-to-peak times over the indicated number of cycles. This curve is expected to decrease to the true value in proportion to one over the square root of the number of cycles used. The dashed line shows the ANOVA prediction of the true value of the standard deviation in period among the fibroblasts. Note that if all cells had the same intrinsic period, and variability of observed period was only due to stochastic fluctuations, then we would expect this graph to approach zero, rather than having a positive horizontal asymptote.

**Figure 3:**
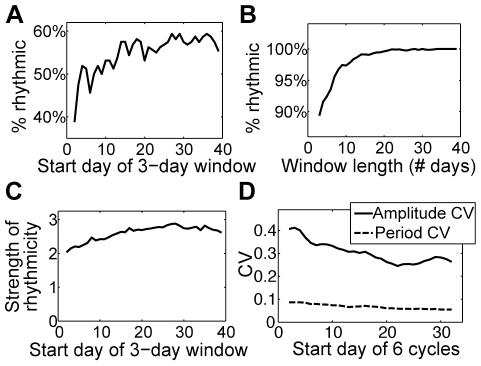
Assessment of cell rhythmicity. (A) Percent of cells with rhythmic 3-day windows is greater for later start times. (B) Percent of rhythmic windows increases with length of window, combining over all cells with start times spaced every 12 h. (C) Strength of rhythmicity (new metric described in Results) of 3-day windows increases over time. (D) CVs of period and amplitude decrease over time, measured using 6 consecutive peak-to-peak times and peak-to-trough amplitudes starting at the indicated day.

All 80 fibroblasts examined were highly rhythmic (autocorrelation: p<0.001 for all time series using 3^rd^ peak of correlogram as described in [18]). See Fig. 1 for examples of PER2::LUC time series, Table 1 for statistics of rhythm parameters, Fig. 2A for a histogram of cell periods, and Fig. 2B for a raster plot comparing 2 cell time-courses. SI Dataset 1 includes time series for all 80 fibroblasts, and Video S1 shows a PER2::LUC bioluminescence recording for one of the cultures. We developed a new test for circadian rhythmicity, described below, to study how the proportion of rhythmic cells depends on the interval length. The percentage of significantly rhythmic 3-day windows (n = 38 per cell for most cells) has a median value of 92% across cells. All cells have at least 61% of 3-day windows rhythmic, but only 10 of the 80 cells are significantly rhythmic in all 3-day windows. The proportion of significantly rhythmic cells increases as the test interval lengthens, with 100% rhythmic for length 21 days or greater (Fig. 3B). Thus, stochastic fluctuations and occasional pauses of oscillations (as in Fig. 1C) can result in short time series segments appearing non-rhythmic, while longer segments present a more complete picture of a cell's intrinsic rhythmicity.

**Table 1:** Summary of descriptive statistics of the 6-week-long fibroblast recordings (*n* = 80 cells from 2 cultures).

	Mean±SD
Period (MESA)	25.02±0.87 h
Period (mean peak-to-peak time)	24.93±0.87 h
Period CV (peak-to-peak times)	0.074±0.023 (Median 0.073)
Amplitude of cells (mean peak-to-trough distance)	3.56±1.69 photons/min
Amplitude CV (peak-to-trough distances)	0.39±0.12 (Median 0.37)
Brightness	2.38±0.94 photons/min
Image area of cells	9250±2120 µm^2^

After eliminating several days at either end of the data series to avoid edge effects and initial transients, 34 cycles were used for calculating cycle length and amplitude statistics of each cell. The period of each cell's time series was calculated using maximum entropy spectral analysis (MESA) and also as mean peak-to-peak time. Coefficient of variation (CV), a dimensionless measure of variability, equals the standard deviation divided by the mean. Brightness (photons/min) equals mean intensity over 34 days of recording.

### Cell rhythms are not affected by position in culture

The cells came from two separate cultures, which are not significantly different in period (p = 0.60) or amplitude (p = 0.13). Coefficient of variation (CV) differs between the two cultures for period (p = 0.01) but not for amplitude (p = 0.43). Cells within each culture appear uncoupled, and there are no significant effects of position within each culture. There was slight clustering of phase at the beginning of recording for each culture (Rayleigh's test, p = 0.06, 0.09), but peak phases were uniformly distributed by the end of recording (p = 0.48, 0.48). There was no significant correlation between period and x-position (p = 0.14, 0.41) or y-position (p = 0.59, 0.85) in the image frame. Looking at all possible pairs of cells (n = 435, 1225 pairs), there was no significant correlation between cell-cell distance and difference in period (p = 0.58, 0.53), in start phase (p = 0.78, 0.27), or in end phase (p = 0.07, 0.86). Restricting distance to 500 microns or less (n = 28, 62 pairs) still did not lead to significant correlations (p = 0.32, 0.11; p = 0.69, 0.76; p = 0.89, 0.58, respectively). We conclude that cells oscillated independently: rhythms were not affected by spatial position in culture, and no coupling was observed between cells. We also judged the two cultures sufficiently similar to combine for statistical analysis of rhythm parameters.

### Fibroblast cell periods are heterogeneous

Circadian periods of individual fibroblasts ranged between 22.5 and 27.0 h (24.93±0.87 h, mean±SD, mean peak-to-peak times, Fig. 2A). We used a random effects model to show that this represents a statistically significant variation of period across cells (ANOVA, F = 6.35, p<0.001). This model assumes that peak-to-peak interval times are distributed as 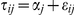, where 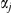 is the mean period of cell *j*, and 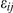 is normally distributed, independent random noise for cycle *i*, cell *j*, that accounts for the variability of peak-to-peak times within each cell. Examination of the data indicates that the assumption of independent, normally distributed values is reasonable. According to the ANOVA results, the standard deviation of the cell periods 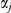 is 0.77 h with 95% confidence interval [0.64,0.94], while the within-cell standard deviation is 1.96 h with 95% confidence interval [1.91,2.02]. This analysis allows us to distinguish the differences in intrinsic period across the population of cells from the variation in period due to stochastic fluctuations within cells. The 6-week length of the recording facilitated accurate evaluation of the heterogeneity, showing that there is variability in period among cells, though it is typically smaller than the variability within cells. See Fig. 2B for an example of two cells with clearly different periods. Fig. 2C shows how the standard deviation in mean cell period changes with the number of cycles, asymptotically approaching the between-cell standard deviation, again emphasizing the importance of a sufficiently long time series.

### Over time in culture, period and amplitude are stable, and both precision and strength of rhythmicity increase

**Figure 4:**
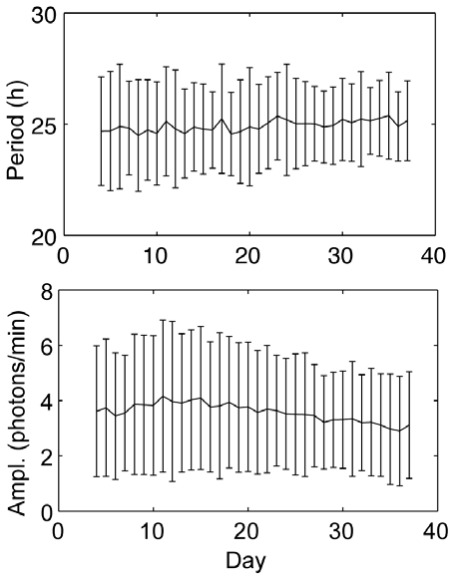
Period and amplitude are remarkably stable over time. Error bars indicate mean±SD across the 80 cells on each day. The mean period changes very gradually, with a slight upward slope of 0.02 hours/day (F = 15,p<0.001), an average change of 0.1% per day. The mean amplitude decreases with a slope of −0.04 photons/min/day (F = 60,p<0.001), an average change of about 1% per day.

We now consider cell periods and amplitudes averaged over the ensemble on different days of the recording, to test the stability over time. Given that there is no apparent coupling between cells, we assume independence between the cells and perform this population averaging to infer single cell characteristics over time. We find that the mean period of the 80 cells is remarkably stable over time, with a slight upward trend of 0.02 hours/day (F = 15, p<0.001), a change of only 0.1% in the period per day (Fig. 4). Thus, each cellular clock likely has a very stable intrinsic period around which cycle lengths fluctuate from day to day. The mean amplitude decreases with a slope of −0.04 photons/min/day (F = 60, p<0.001), an average change of about 1% per day, while the mean brightness decreases with a slope of −0.02 photons/min/day (F = 355, p<0.001), an average change of about 0.7% per day, suggesting that the condition of the cells remained stable during the 6 weeks of recording. CVs of period and amplitude both decrease over time an average of 1.5% per cycle (period CV: slope of regression line −0.001, F = 184, p<0.001; amplitude CV: slope −0.004, F = 248, p<0.001), while strength of rhythmicity (using the new metric, below) increases an average of 0.7% per cycle (slope of regression line is 0.017, F = 196, p<0.001), demonstrating the long-term robustness of the fibroblast oscillators (Fig. 3C and D). Consistent with this improvement in strength of rhythmicity over time is the finding that the proportion of cells with rhythmic 3-day windows increases over time (Fig. 3A). Also observe that the standard deviations shown in Fig. 4 decrease over time, which again is consistent with an increase in precision during the recording, while the mean period remains steady over time.

### Single-cell period is remarkably stable and varies less than amplitude

The coefficients of variation in cycle length for individual cells over time are much lower than those in amplitude (Table 1, Fig. 3D). Another measure of precision, the half-life of the autocorrelation sequence (ACS), confirms the stability of oscillations. To quantify the robustness of oscillations with respect to noise, we divide the ACS half-life by the period to yield a dimensionless parameter that equals number of cycles for the ACS to decrease by 50%. The median value of this parameter is 8.4 cycles, with 1^st^ and 3^rd^ quartiles at 5.1 and 13.8 cycles, respectively.

### Stability of period and amplitude are correlated with cell size and amplitude, but not with period

**Figure 5:**
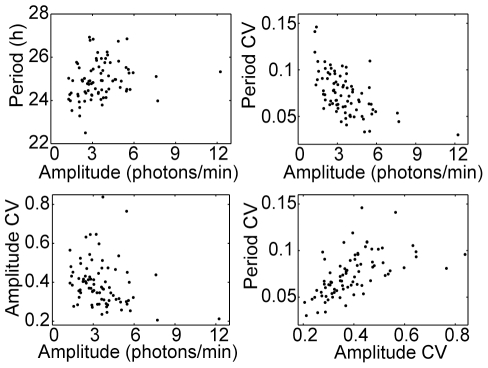
Comparison of cell parameters. Parameters are defined in the caption to Table 1, with correlation coefficients given in Table 2. Greater amplitude tends to be associated with reduced variability, and variability in period tends to be much less than that in amplitude.

This ACS measure of precision is significantly correlated with amplitude (r = 0.37, p<0.001) but not with period (p = 0.95). According to molecular circadian clock models, the ACS half-life should be proportional to cell volume [8], and in fact we do see a positive correlation for the fibroblasts, using the cell area raised to the power 1.5 as a proxy for volume (r = 0.39, p<0.001). Larger cells and those with higher amplitude rhythms are also significantly less variable using standard CV measures, whereas rhythm period is not significantly correlated with period CV or amplitude CV (Table 2, Fig. 5). Clocks in larger cells may involve larger numbers of molecules, thereby reducing variability [8]. For all 80 cells the signal-to-noise ratio (SNR) is strong, averaging 6.49±2.14 (mean±SD), where the SNR equals 10 times the base-10 logarithm of the ratio of the sum of squared signal (circadian component) values to the sum of squared noise values. The SNR is significantly correlated with cell size (r = 0.58, p<0.001) and with brightness (r = 0.72, p<0.001).

**Table 2:** Correlations among fibroblast parameters, as defined in Table 1.

	Period CV	Amplitude	Amplitude CV	Cell size
Period	p = 0.33	*r* = 0.22, p = 0.05	p = 0.66	p = 0.22
Period CV		*r* = −0.58, p<0.001	*r* = 0.57, p<0.001	*r* = −0.46, p<0.001
Amplitude			*r* = −0.26, p = 0.02	*r* = 0.70, p<0.001
Amplitude CV				*r* = −0.30, p = 0.007

Pearson correlation coefficients were calculated; Spearman correlation coefficients gave very similar results.

### Fibroblasts exhibit heterogeneity in a novel metric for strength of rhythmicity

**Figure 6:**
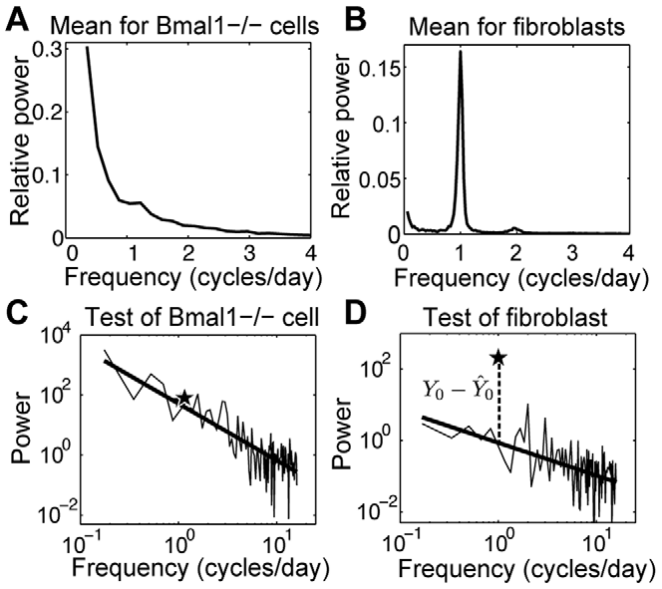
Illustration of new rhythmicity metric. (A) Normalized power spectral density (PSD) averaged across 115 *Bmal1−/−* cell 6-day-long PER2::LUC recordings from Ko et al. [19]. The PSD exhibits characteristics of Brownian noise, with mean slope of the regression line after logarithmic transformation equal to −2.00±0.24 (mean±SD). (B) Averaged PSD for our 80 wild-type fibroblasts using 6-day windows. (C, D) Log-log plot of the PSD and its regression line to illustrate the new rhythmicity test. A star indicates the peak circadian value *Y*_0_ (omitted from the data curve and regression calculation so that we can test whether this value is significantly above the regression line corresponding to background noise), the value on the regression line at the same frequency is 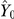, and the dashed line shows the test metric 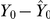. The *Bmal1−/−* cell (C) is judged arrhythmic with 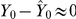, whereas the wild type fibroblast (D) is significantly rhythmic.

To quantify strength of rhythmicity, we develop a novel metric based on the observation that PER2::LUC bioluminescence recordings of arrhythmic *Bmal1−/−* SCN cells from Ko et al. [19] exhibit characteristics consistent with Brownian noise, having a power spectrum proportional to the reciprocal of the frequency squared (Fig. 6A), quite different from the assumption of white noise implicit in many existing rhythmicity tests, e.g., Fisher's *g*-statistic [20]. Hence we propose a rhythmicity criterion testing whether the power associated with the peak circadian frequency is significantly larger than is consistent with an assumption of the power spectrum being proportional to the reciprocal of the frequency to some positive exponent. We take the logarithm of the discrete Fourier transform (DFT) coefficients [21] corresponding to periods between 1.5 h and 120 h, and perform a linear regression to obtain the slope and intercept of the best-fit line as well as the variance of the error. When performing the regression, we omit the DFT coefficient corresponding to the peak circadian period (range 20–30 h), so that we can treat it as a new observation *Y*_0_ and compare its value to the predicted value 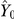 on the regression line (Fig. 6). The difference 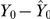 is normally distributed with mean 0 and variance of prediction
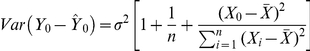
where *X_i_* are the logarithms of the DFT coefficients and *n* is the number of observations used in the regression [22]. We use this to obtain a p-value that measures whether the peak circadian DFT coefficient is consistent with the rest of the data. A one-sided test is used, as we are only interested in whether the peak circadian coefficient is significantly larger than would be expected for arrhythmic cells according to the regression statistics. Because we are testing the rhythmicity of hundreds of windows, we apply a procedure to minimize the false discovery rate [20]: arrange the p-values in ascending order *p*_(1)_,…,*p*_(*N*)_; find the largest *i* for which *p*_(*i*)_≤α*i*/*N*; reject the null hypothesis (that the coefficient is consistent with the regression line for the logarithm of the power spectrum of the noise) for cells corresponding to *p*_(1)_,…,*p*_(*i*)_. We use α = 0.05/*n_f_*, where *n_f_* is the number of DFT frequencies in the circadian range, as a Bonferroni correction for selecting the maximum value among that set.

We use the number of standard deviations by which the peak circadian frequency coefficient is above the regression line as a measure of *strength of rhythmicity*. Applying this test to successive 3-day windows generates a set of 13 values representing the strength of rhythmicity at different times for each cell, with a mean value of 2.6±0.31 across the combined set of windows for the 80 cells. Note that a value of approximately 2 or greater is required for a window to be judged significantly rhythmic (indicating that the circadian coefficient is at least 2 standard deviations above the regression line). This quantity is positively correlated with amplitude (r = 0.60, p<0.001) and cell area (r = 0.44, p<0.001), and negatively correlated with CV of period (r = −0.71, p<0.001) and CV of amplitude (r = −0.52, p<0.001).

An analysis across cells similar to that done for the cell periods indicates that cells differ in strength of rhythmicity (ANOVA, random effects model, F = 2.9, p<0.001). Given the presence of stochastic fluctuations in the oscillations, we want to know how much of the variability in strength of rhythmicity may be attributed to differences among the cells. ANOVA yields a standard deviation of 0.25 for between-cell differences (95% confidence interval [0.19,0.32]) and of 0.67 for within-cell differences (95% confidence interval [0.64,0.70]). Thus, for rhythm strength as well for period, while there is much within-cell variability, there are also significant differences among cells, i.e., some cells are stronger oscillators than others.

### Lack of negative serial correlation of cycle lengths is consistent with PER2::LUC as a direct measure of clock function

A negative serial correlation of circadian cycle lengths is typically interpreted as reflecting a pacemaker-driven process with a variable lag intervening between clock and output measure, i.e. deviations in cycle length of sloppy output measures are compensated by opposite deviations in the following cycle [23]. The serial correlation for the fibroblast PER2::LUC data, however, is 0.06±0.23 (mean±SD), which is significantly positive (t-test, p = 0.01). Given that the fibroblasts are robustly rhythmic, lack of a negative serial correlation may mean that we are directly measuring the clock itself, rather than a clock-driven output. A single limit cycle oscillator subject to noise generally produces phase diffusion with no negative serial correlation [24], and the fibroblast data are consistent with this scenario.

### Stochastic simulations indicate that the fibroblast intracellular circadian clock operates near a Hopf bifurcation such that noise enhances oscillations

As a final step in our analysis, we want to determine whether the fibroblasts are self-sustained oscillators or damped oscillators with noise-sustained rhythms. Westermark et al. [25] described a method to distinguish between these two possibilities by fitting autocorrelation functions for the two kinds of models to experimental time series and comparing the fits to those for stochastic simulations, but the method yielded inconclusive results for the data they considered. The results of this method applied to our longer fibroblast time series were also inconclusive.

Another method using linear modeling combined with Fourier analysis was successfully applied to the p53 gene expression system, with the conclusion that the p53 feedback loop exhibits noise-sustained oscillations, so that without noise the system would exhibit a steady state [26]. In this study, Geva-Zatorsky et al. fit the root mean square average of the Fourier transforms of time courses from 100 cells to the Fourier transform of a third order linear system. We applied this approach to the 80 fibroblasts. The parameter values of the best-fit curve (R^2^ = 0.98; calculated using the Matlab Curve Fitting Toolbox) lead to a system with one positive eigenvalue and a complex conjugate pair with positive real part and imaginary part corresponding to a period of 25.3 h. Interpreting this result is rather difficult, as the fibroblasts are not increasing in amplitude, as these eigenvalues would indicate. Furthermore, the 95% confidence intervals for most of the parameters are quite wide, so little confidence can be placed in the specific parameter values, beyond the conclusion that there is no evidence of damping in the averaged system. It is possible that the intracellular clock mechanism involves nonlinearities strong enough to preclude a linear analysis.

**Figure 7:**
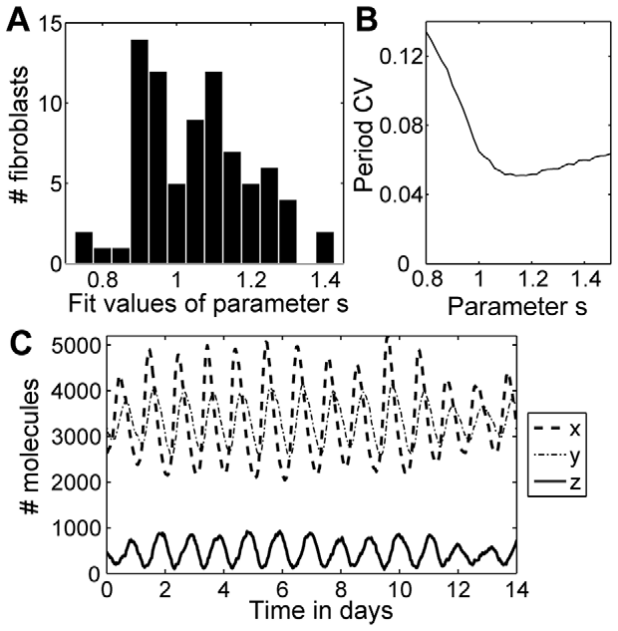
Stochastic modeling. (A) Results from fitting the fibroblast data to the stochastic model, as described in Methods. Fit values with the parameter *s*<1 indicate that the oscillations are noise-induced (the deterministic system would be steady state), while fit values with *s*≥1 indicate that the oscillations are self-sustained. (B) Results of simulations of the stochastic model for different values of the parameter s, fixing the amplitude of *z* to be 500 molecules (by adjusting the value of Ω with respect to *s*). The mean value of the period CV over 500 simulations is shown for each value of *s*. The period CV is minimized for *s* in the range 1.1–1.2. (C) Example of a stochastic simulation with *s* = 1.03, where Ω is chosen so that the amplitude of *z* is 500 molecules. The period CV for this simulation is 0.073.

As an alternative approach, we explored stochastic simulations of a nonlinear model, the modified Goodwin oscillator [27], [28], using the Gillespie algorithm [29], [30], with the goal of determining whether individual cells appeared to be damped or undamped oscillators. See Methods for model details; note that we use a modified system that does not involve high Hill coefficients. This nonlinear model includes the negative feedback loop that is the backbone of the circadian clock, but is simple enough that an explicit expression for the Hopf bifurcation can be derived. The parameter *s* equals 1 at the Hopf bifurcation threshold. Above the Hopf bifurcation (*s*>1) oscillations are self-sustained, while below it (*s*<1) the corresponding deterministic system exhibits damped oscillations but the stochastic system can exhibit noise-induced rhythms. We fit the model to each fibroblast to obtain the parameter value *s* that best reproduces the data (see Methods for fitting procedure). Period variability and amplitude variability for the majority of fibroblasts are best fit by the model when it runs just above the Hopf bifurcation, in the interval *s* = 1.0–1.3, with a minority of cells clustered just below the Hopf bifurcation in the interval *s* = 0.9–1.0 (Fig. 7A). Over the set of 80 fibroblasts, *s* = 1.03±0.14 (mean±SD). Fitting a Gaussian mixture model to the fit parameter values results in two groups, a larger group (81%) centered at *s* = 1.08 and a smaller group (19%) centered at *s* = 0.92.

To explore the possible benefit of particular values of the parameter s, we ran simulations with different values of *s*, where amplitude is fixed to equal the average of the fibroblasts' amplitudes. See Fig. 7C for an example of a simulation with *s* = 1.03 (the average value across the fibroblasts). The simulation results show that the period variability is minimized on the interval *s* = 1.1–1.2 (with minimum of 0.051 at *s* = 1.14), as shown in Fig. 7B. The amplitude CV for the simulations decreases in a sigmoidal fashion as a function of *s*, with amplitude CV around 0.4 for *s*<1, then rapidly decreasing for s>1 to 0.27 at *s* = 1.2, indicating that amplitude variability is always much greater than period variability and that amplitude CV is little changed by moving farther away from the Hopf bifurcation. Therefore the interval of values *s* = 1.0–1.2 may be the most advantageous in terms of minimizing variability. These results suggest that the fibroblast clock is typically a noisy but self-sustained oscillator that operates near a Hopf bifurcation in a range that minimizes stochastic fluctuation of the period. Note that in a deterministic system, running near the Hopf bifurcation is potentially disadvantageous because drifting below *s* = 1 leads to loss of oscillations, but in a stochastic system noise sustains the oscillations so that strong amplitude oscillations can persist.

## Discussion

Our study demonstrates that individual mammalian fibroblast cells, after more than 6 months in vitro, can generate stable, independent circadian oscillations for at least 6 weeks in a constant environment. This is consistent with and builds on previous findings [14], [15] that dissociated fibroblasts in culture are persistent, independent oscillators, with no evidence of damping or functional coupling. All 80 fibroblasts exhibit strong circadian rhythms of PER2::LUC bioluminescence, with 100% of cells significantly rhythmic whether using a standard test such as autocorrelation or the new metric described above. Period and amplitude across the 80 cells are very stable over the 6 weeks, with precision and strength of rhythmicity gradually increasing over time.

Previous studies examining rhythmicity and variability of single-cell circadian rhythms were based on relatively short time series (6–15 days). With 42 days of recording, the present study allows a more complete and detailed analysis. The high proportion of rhythmic 3-day windows (Fig. 3A) indicates that intervals of arrhythmicity (e.g., Fig. 1C) do occur but are relatively rare. The long recordings also allow us to properly assess heterogeneity in the population, which can be overestimated when using short recordings (Fig. 2C). Our results suggest that recording cells for at least 3 weeks is required for accurate assessment of rhythmicity and its variability within and among cells. In particular, our finding that the proportion of rhythmic cells is underestimated for recordings shorter than 3 weeks could explain results in the literature in which cells recorded for relatively short durations exhibit a mixture of rhythmicity and arrhythmicity. Longer recordings are required to determine whether subpopulations of cells are truly arrhythmic.

Although the 80 fibroblasts should be genetically identical and in a constant environment, we find substantial heterogeneity in period and strength of rhythmicity among cells. Because the cells are noisy oscillators, distinguishing within-cell variability from between-cell variability is important, to avoid overestimating the differences among cells. The standard deviation in cell period across the population is 0.77 h, indicating significant though modest differences in period among the cells. The within-cell standard deviation is much greater, 1.96 h, signifying that the cells exhibit significant stochasticity, resulting in cycle-to-cycle variability around each cell's mean period.

Carr and Whitmore [16] suggest that genetically similar cells should exhibit nearly identical free-running periods, and that a wide range of observed periods implies an unstable clock. However, we find that the fibroblast population's mean period is quite stable over time (Fig. 4), and differences among cells could well result from differences in cell size or epigenetic effects [31]. Rhythm parameters were not related to spatial position of a cell, arguing against environmental effects as a cause of heterogeneity, but larger cells did tend to be less variable in both period and amplitude. Geva-Zatorsky et al. [32] observed similarly variable responses in a population of cells known to be isogenic, including large differences in period of oscillation in an oscillatory transcriptional circuit involving the tumor suppressor p53 and the oncogene Mdm2. Substantial differences in gene expression among cells in clonal populations have also been observed in other settings [9], [33].

Period and amplitude fluctuate over time due to the stochastic nature of the molecular clock mechanism, consistent with the long-term fluctuations in levels of diverse proteins observed previously in single cells [34]. Recording noisy oscillators for only a few cycles can yield misleading results, e.g., causing an overestimate of the population's variability, as well as making it difficult to quantify accurately the cycle-to-cycle variability of each cell. Fluctuations during short recordings may also cause some cells to be judged arrhythmic when in reality they are rhythmic. The long recording duration of our study demonstrates the persistent nature of the fibroblasts' clocks and allows a better estimate of their precision. In our fibroblasts, we do not find evidence of the very large fluctuations found in zebrafish cells [16]. This may partly be due to differences in cell type and reporter used, but could also be due to differences in length of recording and analysis methods: the zebrafish study used relatively short recordings, and using FFT-NLLS to assess period in 2-day windows may have overestimated period variability. We compared 6 alternative methods of assessing period to ensure reliability, an approach used successfully in other studies [12], [13].

We find that period is much more stable than amplitude in these fibroblast single-cell circadian oscillators, with a median CV of 7.3% for period and a median CV of 37% for amplitude. A similar pattern was observed in experiments on the p53 oscillator, where stochastic modeling suggests that the relatively higher variability in amplitude is best accounted for by low frequency noise in protein production rates that occurs when an oscillator amplifies noise near its resonant frequency [32]. Noise in protein degradation rates, on the other hand, tends to produce equal variability in period and amplitude. Relatively precise period and more variable amplitude has been observed in experiments and model simulations elsewhere in the literature, leading Geva-Zatorsky et al. [32] to suggest this may be a general feature of biological oscillators; our data support this hypothesis.

Stochastic fluctuations in expression of mammalian genes [9], including circadian clock genes [35], emerge mainly from transcriptional bursting, and this intrinsic noise can contribute to variability in circadian oscillations [7], [8]. Some clocks, e.g., the transcription-independent circadian clock in cyanobacteria [36], may have evolved to be largely independent of oscillations in mRNA in order to minimize this source of noise, resulting in extremely high precision [17]. Interestingly, however, the noise of cyanobacterial clock gene expression varies systematically over the circadian cycle [37].

The fibroblasts' impressive cycle-to-cycle precision (CV 7.3%) is comparable to that of SCN neurons (CV 8.8%) [13], although less than the precision reported for cyanobacteria [17]. Genetic circuits are inherently noisy and so have evolved to function in the presence of fluctuations. The circadian oscillation mechanism may be constrained by the requirement of generating reliable rhythms despite fluctuations due to intrinsic noise [7]. In stochastic models of transcriptional circadian clocks, precision can be improved by increasing cell size or copy number of clock genes (both of which increase the number of molecules involved), increasing rates of binding/unbinding of regulatory proteins at clock gene promoters, or increasing cooperativity of negative feedback [8], [38].

Gene regulatory circuits with different architectures can generate similar dynamics but different noise characteristics [39], and clocks may have evolved specific circuitry to minimize noise. Negative feedback can reduce noise [40], [41], and longer negative feedback pathways could improve precision by averaging out the molecular noise inherent in each step of the pathway [42]. Positive feedback loops may allow buffering against propagated noise while maintaining sensitivity to entraining signals [43]. They speed the transition from low to high transcription, minimizing the portion of a cycle with low transcription rate, which is especially vulnerable to molecular noise [44]. High flux through a long circuit could also allow high precision in a cellular clock under the constraint of low molecule counts [45].

Interestingly, however, under some conditions, biological oscillators may be not only resistant to noise, but actually dependent on intrinsic biochemical noise for sustaining oscillations [44] or for optimal precision [46]. An example is the phenomenon of coherence resonance, which occurs when noise excites coherent oscillations in a system that would otherwise exhibit a steady state, for instance, when a system is near a Hopf bifurcation [47]. For damped oscillators in which damping occurs on a slower time scale than the oscillation, noise can amplify and sustain oscillations through coherence resonance [48]. Furthermore, in coherence resonance there is typically an optimal noise level that maximizes the coherence (regularity) of the oscillations [49]. These findings in the literature suggest that biological oscillators have incorporated multiple mechanisms to optimize rhythms in the unavoidable presence of noise.

In particular, our modeling results suggest that the fibroblast molecular clock operates in an optimal range near a Hopf bifurcation (Fig. 7). According to our model, the optimal parameter values are above the Hopf bifurcation, so the underlying principle is not that of coherence resonance. However, because this optimal parameter range is close to the Hopf bifurcation, one would expect the observed distribution of parameter values to include some that fall below the Hopf bifurcation, in which case intrinsic noise will sustain the amplitude of oscillations. Running just above the Hopf bifurcation in a deterministic system is potentially disadvantageous because drifting below *s* = 1 leads to loss of oscillations. In contrast, in a stochastic system, noise can sustain the oscillations so that high amplitude oscillations persist while allowing the oscillator to run near the Hopf bifurcation where period variability is minimized.

For a given network structure, variability in period of cellular circadian clocks has important implications for synchronization in a multi-oscillator system [50]. SCN neurons connected in a small-world network synchronize efficiently to one another [51], whereas peripheral cells may not synchronize to one another at all, but instead entrain to a common signal emanating from the SCN. The lack of coupling among peripheral oscillator cells makes them more responsive to synchronizing signals, despite substantial cell-to-cell variability of intrinsic free-running period [52]. At the same time, cycle-to-cycle variability may ease entrainment by lowering the coupling strength needed to synchronize, as well as increasing the probability that the period of an errant cell will wander back into the range of entrainment [53], [54]. Thus, consistent with our findings, fibroblast clocks may find it advantageous to have cycle-to-cycle fluctuations in period that are greater than the variability across the cell population. Synchronization also occurs more readily for damped oscillators with noise-sustained oscillations [55], another advantage of operating close to a Hopf bifurcation. Operating in an optimal zone near a Hopf bifurcation threshold may allow peripheral clocks to run stably while remaining sufficiently flexible to easily entrain to pacemaker signals.

## Methods

### Ethics statement

This study was approved by the Institutional Animal Care and Use Committees of The Scripps Research Institute and Unversity of California, San Diego. Every effort was made to minimize the number of animals used, and their suffering.

### Mice

We used mice harboring an *mPer2^Luc^* (PER2::LUC) knockin reporter of circadian clock function [56]. In these mice, the *mPer2* gene is replaced by a reporter gene coding for firefly luciferase, fused to the C-terminus of the wild type mPER2 protein. This PER2::LUC fusion protein is a highly faithful reporter of circadian clock function because its expression reflects the full transcriptional and post-transcriptional regulation of the *mPer2* locus. For this study, we used an alternative PER2::LUC mouse line incorporating an SV40 polyadenylation site to enhance expression levels. Mice were bred as homozygotes on a mixed B6/129 genetic background, and maintained in LD 12∶12 light cycles (12 hrs light, 12 hrs dark) throughout gestation and from birth until used for experiments.

### Cells

Primary fibroblast culture and luminometry were performed as previously described [14], [57]. Fibroblasts were dissociated from tails of neonatal PER2::LUC mice by a standard enzymatic digestion procedure, cultured in high glucose DMEM supplemented with 10% fetal bovine serum (FBS), and grown to confluence. For luminescence recordings, cells were transferred to HEPES-buffered, air-equilibrated DMEM medium (GIBCO 12100-046), supplemented with 10 mM HEPES, 1.2 g/L NaHCO_3_, 25 U/ml penicillin, 25 µg/ml streptomycin, 2% B-27 (GIBCO 17504-044), and 1 mM luciferin (BioSynth L-8220), pH 7.4. The sealed 35 mm culture dishes were then placed into a luminometer (LumiCycle, Actimetrics, Inc.), inside a standard tissue culture incubator kept at 36°C; no CO_2_ was added, and ambient CO_2_ was <0.04%. Luminescence from each dish was measured by a photomultiplier tube for ∼70 sec at intervals of 10 min, and recorded as counts/sec. To select stable cultures for single-cell imaging, we recorded bioluminescence rhythms in the luminometer for at least 6 months, with a medium change every 1–2 weeks. Cells did not divide in serum-free medium, and maintained a density of ∼100 cells/mm^2^. Cultures that continued to generate strong PER2::LUC rhythms after 6 months were used for single-cell imaging experiments.

### Single-Cell Imaging

Long-term single-cell bioluminescence imaging was performed as previously described [14], [57]. Just before imaging, medium was changed to the same medium used for luminometer recording. Culture dishes were sealed and placed on the stage of an inverted microscope (Olympus IX71) in a dark room. A heated lucite chamber around the microscope stage (Solent Scientific, UK) kept the cells at a constant 36°C. Images were collected by an Olympus 4× UPlanApo (NA 0.16) objective and transmitted to a CCD camera (Spectral Instruments SI800, Tucson, AZ). Thermal (dark current) noise was minimized by cooling to −90°C. Read noise was minimized by 8×8 binning of pixels. Images of 29.8 min exposure duration were collected at 30 min intervals for 42–44 days. Integration of bioluminescence over all single cells analyzed or the entire imaging field gave population patterns similar to those measured in the luminometer.

### Single-Cell Image Processing

In MetaMorph (Molecular Devices), cosmic ray artifacts were removed by using the minimum value for each pixel in a pixel-wise comparison of two consecutive images. Thus, data were effectively smoothed by a running minimum algorithm, with a 1 hr temporal window. Images were corrected for bias and dark current by background subtraction. In the resulting stack of images, cells that were clearly discriminable from adjacent cells and remained bioluminescent for the entire experiment were selected for analysis. Luminescence intensity was measured within a region of interest defined manually for each cell. The position of the region was adjusted if necessary to accommodate movements of cells. Data were logged to Microsoft Excel for further processing and plotting. Luminescence intensity values were converted to photons/min based on the rated quantum efficiency and gain of the camera.

### Time series analysis

We applied the translation-invariant discrete wavelet transform [21], [58] to each time series, using custom Matlab scripts incorporating C. Cornish's *wmtsa* package. This transform removes high frequency noise and long-term trend, leaving intact a frequency band corresponding to periods of either 8–64 h or 16–64 h. Two days of filtered signal were discarded at each end of the time series to avoid edge effects. In transformed data leaving the 16–64 h period band intact, phase markers such as peak times could be clearly identified and used to determine cycle lengths. In transformed data leaving the 8–64 h period band intact, amplitude was measured as peak-to-trough height. We compared 6 measures of cycle lengths: time between successive peaks, troughs, upward or downward midpoint crossings of the wavelet transformed data; time between successive zero phases of the Hilbert transformed time series (using the *hilbert* function in the Matlab Signal Processing Toolbox); as well as instantaneous period estimated by the analytic wavelet transform ridge (using *jlab* Version 0.91 by J. Lilly). Estimates of mean period and period variability were highly consistent across these measures, except that the analytic wavelet transform yielded consistently smaller variability estimates due to effectively using a weighted 3-day window, whereas the other methods used single cycles. We chose to use peak times to estimate cycle lengths, a method used in many other studies. Scripts were run on Matlab 7.13.0 (Mathworks, Natick, MA).

### Stochastic modeling

Stochastic simulations of the modified Goodwin oscillator (converted to mass action kinetics as described in [8]) using the Gillespie algorithm were generated using StochKit2 [30]. The differential equations for the deterministic system are
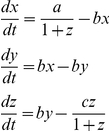
The variable *z*, subsampled at 0.5 h intervals, is used for statistical analysis to mimic the experimental data. See [27] for a derivation of the expression for the Hopf bifurcation point in terms of parameters *a*, *b*, and *c*, all assumed to have positive values. To summarize, under the simplifying assumption 
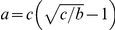
, the stationary point is 
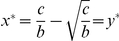
, 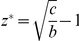, and a Hopf bifurcation occurs when *c* = 81*b*, switching the dynamics from a stable steady state for *c*<81*b* to stable oscillations for *c*>81*b*, in which case the period is approximately 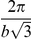. For convenience, we define the parameter 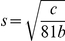 so that *s*<1 indicates a stable steady state and *s*>1 indicates stable oscillations in the deterministic system. In the stochastic version of this system with volume parameter Ω(used to convert concentrations *x*,*y*,*z* to numbers of molecules *X*,*Y*,*Z*: *x* = *X*/Ω, *y* = *Y*/Ω, *z* = *Z*/Ω), noise can excite coherent oscillations even when *s*<1. We fit the parameter *s* for each fibroblast using a maximum likelihood measure on the period and amplitude variability, where *b* is chosen to match the cell's mean period and Ω is chosen in coordination with *s* to ensure that the simulated mean amplitude agrees with the experimental value. We converted amplitude in photons/min to molecules by multiplying by 500/3.56, so that a cell with average amplitude was simulated with an amplitude of 500 in the *z*-variable (the peak number of molecules typically being considerably larger than the amplitude value, which is the difference between peak and nadir values; peak values for *x* and *y* tended to be 10 times greater than the amplitude of *z*). This choice yielded excellent fits and reproduced the important features of the experimental data. Increasing this ratio by 20% led to a less than 2% change in the fit values for *s*. Gaussian mixture model fits were done using Matlab's gmdistribution.fit, applying the Akaike Information Criterion to determine the number of components.

## Supporting Information

Dataset S1 **Spreadsheet with bioluminescence data in photons/min for the 80 fibroblasts.**
(CSV)

Video S1 **Circadian Rhythms of Fibroblasts Persist for Six Weeks.** Time-lapse bioluminescence movie of primary fibroblasts from PER2::LUC mice recorded for 44 days under constant conditions. Original 29.8 min exposures were collected at 30 min intervals, with 8×8 binning of pixels to reduce read noise. Cosmic ray artifacts were removed, and a background image was subtracted (see Methods). The field of view is 3.3 mm across, and the duration of the movie is 43.9 days. Note the regularly oscillating cells, many of which remain rhythmic over the entire six weeks. The culture shown here contains cells #1–30 of the 80 cells selected from 2 cultures for quantitative analysis.
(MOV)
